# The synergistic effects of oxaliplatin and piperlongumine on colorectal cancer are mediated by oxidative stress

**DOI:** 10.1038/s41419-019-1824-6

**Published:** 2019-08-08

**Authors:** WeiQian Chen, WeiShuai Lian, YiFeng Yuan, MaoQuan Li

**Affiliations:** Department of Intervention and Vascular Surgery, Shanghai Tenth People’s Hospital, Tongji University School of Medicine, Shanghai, China

**Keywords:** Chemotherapy, Targeted therapies

## Abstract

Oxaliplatin-based chemotherapy is recommended as the first-line therapeutic regimen for metastatic colorectal cancer. However, long-term and repeated oxaliplatin therapy leads to drug resistance and severe adverse events, which hamper its clinical application. Thus, chemosensitizers are urgently required for overcoming oxaliplatin resistance and toxicity. Here, the anticancer effects of oxaliplatin combined with piperlongumine (PL), a molecule promoting reactive oxygen species (ROS) generation, in colorectal cancer, were assessed. We demonstrated that oxaliplatin elevated cellular ROS amounts and showed synergistic anticancer effects with PL in colorectal cancer cells. These anticancer effects were mediated by mitochondrial dysfunction and endoplasmic reticulum (ER) stress apoptotic-associated networks. Meanwhile, blockage of ROS production prevented apoptosis and fully reversed mitochondrial dysfunction and ER stress associated with the oxaliplatin/PL combination. Moreover, xenograft assays in mouse models highly corroborated in vitro data. In conclusion, this study provides a novel combination therapy for colorectal cancer, and reveals that manipulating ROS production might constitute an effective tool for developing novel treatments in colorectal cancer.

## Introduction

Colorectal cancer (CRC) represents the third deadliest malignancy in the West^1^. Currently, the only curative treatment is surgical resection. However, timely diagnosis is difficult, as early signs are either unspecific or insignificant. The majority of colorectal malignancy cases are detected at advanced stages. Treatment prognosis in early-stage CRC is generally quite promising, with an ~90% cure rate, which drops to below 10% in patients with advanced-stage CRC. Targeted chemotherapy and chemotherapy are frequently used in CRC management, and known to improve long-term survival and reduce recurrence. Conventional chemotherapy, on the other hand, has a limited effect and causes detrimental side effects to patients, due to a lack of specificity. Therefore, a new drug or method is necessary for improving prognosis.

Oxaliplatin was the first platinum drug with proven activity against CRC, and has been considered the standard treatment in CRC. The underlying mechanism is probably associated with cell death induced by platinum–DNA abducts, and stronger inhibitory effects on DNA replication are observed with oxaliplatin, compared with other platinum agents, such as cisplatin^2^. However, oxaliplatin was reported by the Food and Drug Administration to be responsible for serious adverse events, such as gastrointestinal, hematological, and neurological toxicities^2^, often leading to treatment discontinuation^3^. In addition, these serious adverse events in patients receiving oxaliplatin are the principal dose-limiting toxicities^2–4^. Hence, oxaliplatin combination with other chemotherapeutics has been proposed for improving the efficacy and safety in clinical settings.

Reactive oxygen species (ROS) are formed as natural by-products in various cellular activities, such as protein folding and mitochondrial metabolism^5^. Studies have shown that malignant cells have higher endogenous oxidative stress in comparison with nonmalignant counterparts, which has been attributed to imbalanced redox status^5,6^. Therefore, malignant cells are more intolerant of additional oxidative stress compared with noncancerous cells, due to higher baseline oxidative stress levels. This makes malignant cells sensitive to agents that raise ROS levels^7^. Recent studies have shown that ROS generation in malignant cells constitutes a mechanism underpinning the synergetic cytotoxic effects observed with combinatory anticancer treatments^8,9^. Meanwhile, platinum-based compounds were reported to induce cytotoxicity via oxidative stress^10–12^, and may lead to ROS generation both directly and indirectly^13,14^. Our previous study demonstrated that EF24, an ROS inducer, increases gastric cancer cell sensitivity to rapamycin in an ROS-dependent manner^15^. Therefore, new combinatory therapeutic regimens targeting ROS could further improve patient outcome in CRC.

In this study, we discovered an ROS-targeted combinatory treatment for CRC, comprising oxaliplatin and the natural product piperlongumine (PL). PL is considered a direct TrxR1 inhibitor, which kills malignant cells in an ROS-dependent fashion^16^. This work showed that PL enhances CRC cell sensitivity to oxaliplatin in vitro via induction of an ROS-dependent mitochondrial dysfunction and endoplasmic reticulum (ER) stress apoptotic pathways, and blocking ROS synthesis by a specific inhibitor fully blunted the synergistic antitumor activity. In addition, we showed that oxaliplatin displays synergistic effects with PL on CRC in vivo. These findings indicate that, oxaliplatin combined with ROS-inducing agents, might constitute an efficient alternative for CRC treatment in the clinical setting.

## Materials and methods

### Cell culture and reagents

Human CRC (HCT-116 and LoVo) and noncancerous gastric epithelial (GES-1) cells were provided by the Institute of Biochemistry and Cell Biology, Chinese Academy of Sciences (China), and maintained in DMEM containing 10% heat-inactivated fetal bovine serum (FBS), and 100 U/mL penicillin and 100 μg/mL streptomycin (Gibco, USA). Oxaliplatin and piperlongumine were provided by Selleck Chemical (China). *N*-acetyl-l-cysteine (NAC) was manufactured by Beyotime Biotech (China). Antibodies targeting Bax, cleaved poly-ADP ribose polymerase (PARP), Bcl-2, phosphorylated histone 2AX (γH2AX), ATF4 (activating transcription factor-4), CHOP (CCAAT/enhancer-binding protein homologous protein), p-eIF2α, and eIF2α (eukaryotic initiating factor 2) were manufactured by Cell Signaling (USA). Anti-GAPDH primary and horseradish peroxidase-conjugated secondary antibodies were provided by Santa Cruz (USA). FITC-Annexin V apoptosis Detection Kit I and propidium iodide (PI) were manufactured by BD Pharmingen (USA).

### Cell viability assay

The effects of oxaliplatin and PL on cell viability were assessed by the 3-(4,5-dimethyl thiazol-2-yl)-2,5-diphenyl tetrazolium bromide (MTT) assay. Cells in 96-well plates at 5000–10,000/well underwent overnight incubation. Oxaliplatin at the final concentrations of 0.3125, 0.625, 1.25, 2.5, 5, 10, 20, 50, 75, and 100 μM, respectively, was added as single treatment or in combination with PL at a fixed concentration. Incubation was performed for 24 h followed by the MTT assay. Next, PL at the final concentrations of 0.625, 1.25, 2.5, 5, 10, 15, 20, 30, and 50 μM, respectively, was added to cells as single treatment for 24 h, followed by the MTT assay. Both oxaliplatin and PL were dissolved in DMSO with further dilution in DMEM. The IC_50_ values were assessed by the Logit technique.

### Cell apoptosis assessment

For apoptosis detection, cells were plated at 3 × 10^5^/well in six-well plates for overnight incubation followed by administration of oxaliplatin and/or PL, for 24 h with or without 5 mM NAC pretreatment. Then, the treated cells underwent PBS washes before evaluation for apoptosis, using Annexin V and PI double staining. Each group was evaluated on a FACS Calibur flow cytometer.

### Caspase-3 activity measurement

Caspase-3 activity in cell lysates was assessed with the Caspase-3 activity kit, strictly following the protocol provided by the manufacturer.

### Intracellular oxidant detection

Cellular oxidant production was assessed flow-cytometrically as described previously^16^. In brief, 5 × 10^5^ cells/well in six-well plates underwent overnight incubation in normal growth medium. This was followed by administration of oxaliplatin and/or PL, at concentrations and times indicated. NAC pretreatment was performed for 2 h in NAC groups. Upon treatment, cells underwent incubation (30 min at 37 °C away from light) with the ROS indicator DCFHDA (10 μM) in medium without FBS for the detection of ROS. After PBS washes, fluorescence was assessed flow-cytometrically on a FACS Calibur. Alternatively, cells were seeded on glass slides and incubated with DCFHDA for ROS level assessment under a Nikon epifluorescence microscope.

### Western blot

Cells were lysed with cell lysis buffer and the lysates were centrifuged (12,000 rpm, 10 min at 4° C) to remove the debris. Total protein amounts were obtained by the Lowry method. Equal amounts of protein were mixed with sample loading buffer before electrophoresis. Once electrophoresis was completed, the bands were transferred onto polyvinylidene difluoride (PVDF) transfer membranes. The membranes were subsequently blocked with 5% fat free milk in TBST buffer (10 mM Tris-HCl, pH 7.4, 150 mM NaCl, 0.1% Tween 20) for 2 h in ambient conditions. After washing, the samples underwent incubation with specific antibodies overnight at 4 °C, and subsequently developed with HRP-linked secondary antibodies for 1 h. Visualization was performed with the ECL kit (Bio-Rad, USA). Image J (National Institute of Health, USA) was used to quantify the intensities of the immunoreactive bands.

### ER electron microscopy

HCT-116 cells were administered DMSO or oxaliplatin in combination with PL after pretreatment or not with 5 mM NAC for indicated times. After treatment, cells underwent fixation with 2.5% glutaraldehyde in PBS overnight at 4 °C and postfixation with 1% OsO_4_ in ambient conditions for 1 h. Following the fixation procedure, cells were stained with 1% uranyl acetate, and underwent dehydration (graded acetone solutions) and Epon embedding. Finally, 70 nm sections were obtained and assessed under an H-7500 electron microscope (Hitachi, Japan).

### Assessment of mitochondrial membrane potential (Δψm) change

JC-1 is readily taken up by cells and healthy mitochondria. The JC-1 probe (green fluorescence) is a monomer at low membrane potential, but yields ‘J-aggregates’ (red fluorescence) at elevated membrane potential. The red/green fluorescence ratio depends solely on mitochondrial membrane potential and is not associated with other parameters potentially affecting fluorescence, including mitochondrial size, shape, and density^17^. Briefly, the cells were administered oxaliplatin and/or PL for 12 h, followed by incubation (37 °C, 1 h) with 5 mg/l JC-1 (Beyotime Biotech). NAC pretreatment (as indicated) was performed for 2 h. Following the incubation, cells underwent PBS washes and were resuspended in serum-free medium. Finally, a Nikon epifluorescence microscope equipped with a digital camera (Nikon, Japan) was employed for imaging (green, excitation, 490 nm and emission, 530 nm; red, excitation, 540 nm and emission, 590 nm).

### In vivo xenograft model

Experiments involving animals fully complied with the ARRIVE guidelines^18^. The animals were housed in individual cages in the Animal Facility of Tongji University. The assays were performed following the National Institutes of Health Guide for the Care and Use of Laboratory Animals, and had approval from the Biological Research Ethics Committee of the Chinese Academy of Sciences. Five-week-old female athymic BALB/c nu/nu mice (between 18 and 22 g) obtained from SLAC Laboratory Animal Co., Ltd (China) were maintained at 26 ± 1 °C under a 12 h/12 h light–dark cycle, with standard rodent chow and water freely available. HCT-116 cells (10^7^ in 150 μL PBS) were administered subcutaneously into the animal’s right flank. After the xenografts grew to 50–100 mm^3^ (about 8 days), the animals were intraperitoneally administered 5 mg/kg oxaliplatin and/or 2.5 mg/kg PL once daily. At various time points (up to 24 days), euthanasia was performed under anesthesia with i.p. pentobarbital sodium (50 mg/kg) followed by tumor harvest. The tumors were assessed for weight and volume [V = (width)^2^ × length/2]. In addition, specimens were treated for histological assessment and immunoblot.

### Statistical analysis

Data analysis was performed as previously recommended for similar studies^19^. Data are expressed mean ± SEM. GraphPad Pro. Prism 5.0 (GraphPad, CA) was employed to analyze the data, which were compared by unpaired *t*-test. *P* < 0.05 indicated statistical significance.

## Results

### High oxaliplatin levels suppress the growth of colorectal cancer and noncancerous cells without excellent selectivity

**Fig. 1:**
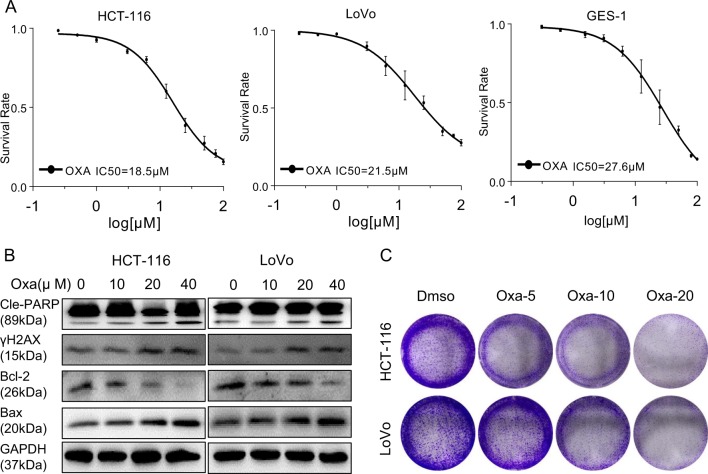
Oxaliplatin causes cytotoxicity in human colorectal cancer and noncancerous cells. **a** Oxaliplatin reduced colorectal cancer cell viability. HCT-116, LoVo, and GES-1 cells were administered increasing oxaliplatin amounts (0.25–100 μM) for 24 h, and cell viability was measured by the MTT assay. **b** Colorectal cancer cells were administered oxaliplatin (10, 20, or 40 μM) for 16 h, and apoptosis and DNA repair-related proteins were assessed by immunoblot. **c** Effect of oxaliplatin (5, 10, or 20 μM) on colorectal cancer cell colony formation. Cells were stained with crystal violet after 14 days of treatment

To assess oxaliplatin’s effects on CRC and noncancerous gastric cell proliferation, oxaliplatin was administered at various concentrations for 24 h and viability was quantitated by the MTT assay. The results showed that oxaliplatin reduced viability in HCT-116, LoVo, and noncancerous GES-1 cells with IC_50_ values of 18.5, 21.5, and 27.6 μM, respectively (Fig. 1a). These findings indicated that oxaliplatin at high levels indiscriminately killed both cancer and noncancerous cells. At safe levels (nontoxic to GES-1 cells), oxaliplatin displayed limited inhibitory effects on CRC cells. Whether oxaliplatin reduced viability via apoptosis induction was next assessed. As oxaliplatin is thought to trigger cell apoptosis mainly by inducing DNA damage, the levels of DNA repair and apoptosis-associated proteins in HCT-116 and LoVo cells were quantitated. The levels of phosphorylated histone 2AX (γH2AX) and cleaved PARP were assessed as indexes of DNA damage and apoptosis, respectively^20,21^. As shown in Fig. 1b, the amounts of Bax, cleaved PARP, and phosphorylated histone 2AX (γH2AX) were elevated, while Bcl-2 was less abundant after oxaliplatin administration. These effects were concentration-dependent, with pronounced DNA damage and apoptosis at oxaliplatin concentration above 20 μM. Moreover, the colony formation assay confirmed that oxaliplatin dose-dependently decreased viability in human CRC cells, significantly at 20 μM oxaliplatin (Fig. 1c).

### Piperlongumine (PL) sensitizes human colorectal cancer cells to oxaliplatin, and enhances oxaliplatin-associated ROS production

**Fig. 2:**
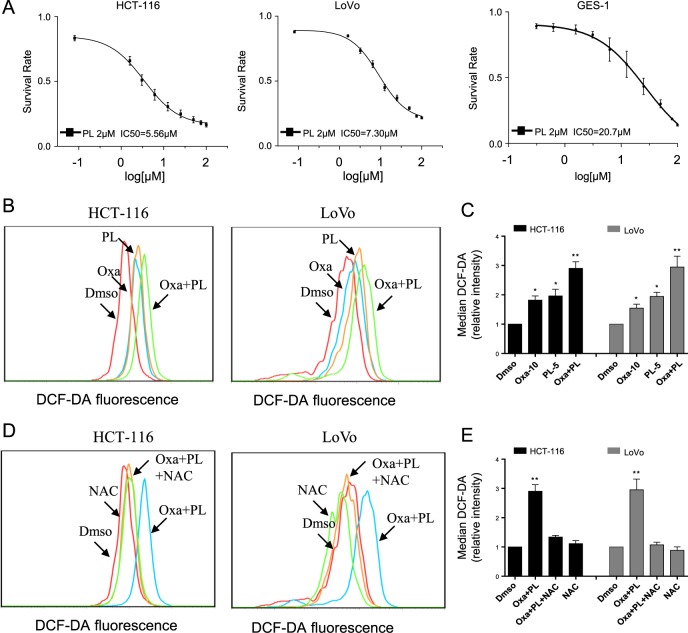
PL enhances oxaliplatin-induced anticancer effects and ROS production in colorectal cancer cells. (**a**) HCT-116, LoVo and GES-1 cells underwent pretreatment with 2 μM PL and further administered increasing doses of oxaliplatin (0.25–100 μM) for 24 h. Cell viability was measured by the MTT assay. (**b**) PL enhanced oxaliplatin-associated ROS generation in HCT-116 and LoVo cells. Cells were administered PL and/or oxaliplatin for 1 h, and then intracellular ROS amounts were measured using DCFHDA by flow cytometry. (**c**) Quantification of DCF data in (**b**). (**d**) Pretreatment of cells with NAC completely blunted ROS production. HCT-116 and LoVo cells were pretreated with 5 mM NAC for 2 h before combined treatment with oxaliplatin (10 μM) and PL (5 μM). ROS production was assessed flow-cytometrically. (**e**) Quantification of DCF data in (**d**) Data are mean ± SEM and were assessed by Student’s t-test. *P < 0.05 vs. DMSO control

Due to its unspecific nature, chemotherapy is generally given at low dose to minimize systemic cytotoxicity. As a result, chemotherapy research has been focusing on combination therapies, which have the potential to generate high levels of cancer cell cytotoxicity at low concentrations. It has been shown that PL, a compound naturally occurring in the long pepper *Piper longum* L, exhibits suppressive activity against cancer and increases cancer cell sensitivity to anticancer drugs via many mechanisms^16,22–24^. Hence, we hypothesized that PL could enhance oxaliplatin’s antitumor properties in human CRC. In this study, CRC cells were administered increasing amounts of oxaliplatin combined with 2 μM PL for 24 h. At a concentration of 2.0 μM, PL as monotherapy did not inhibit HCT-116 or LoVo cells remarkably (Supplementary Fig. S1A). However, the above combination treatment markedly decreased cancer cell viability in comparison with the monotherapies, but did not affect normal cells (IC_50_ values of 5.56, 7.30, and 20.7 μM for HCT-116, LoVo, and GES-1 cells, respectively (Fig. 2a). It is admitted that elevated ROS amounts in malignant cells may explain synergetic cytotoxic effects observed with select antitumor treatments^15,22^. Meanwhile, platinum-based compounds have been reported to enhance intracellular ROS levels in ovarian and bladder cancer cells^10,25^. Therefore, whether ROS generation was involved in the synergistic anticancer effects of oxaliplatin and PL was assessed. ROS amounts in both CRC cell lines were assessed using DCFHDA, which is deacetylated and oxidized to DCF by ROS. Treatment of HCT-116 cells with oxaliplatin (30 μM) alone dose-dependently increased intracellular ROS levels, approximately reaching the peak at 1 h (Fig. S2A). Treatment of cells with oxaliplatin alone for 1 h dose dependently increased ROS production (Figs. S2B, C). Co-treatment of cells with oxaliplatin (10 μM) and 5 μM PL resulted in the significantly higher ROS amounts in comparison with oxaliplatin or PL monotherapy (Fig. 2b, c and S2D). As expected, elevated DCF signals (ROS amounts) were absent after pretreatment with the ROS scavenger *N*-acetyl cysteine (NAC) for 2 h (Fig. 2d, e and S2D). Taken together, the above findings suggested that intracellular ROS production played a vital role in the synergetic anticancer effects of oxaliplatin and PL.

### PL enhances oxaliplatin-induced apoptosis in a ROS-dependent fashion

**Fig. 3:**
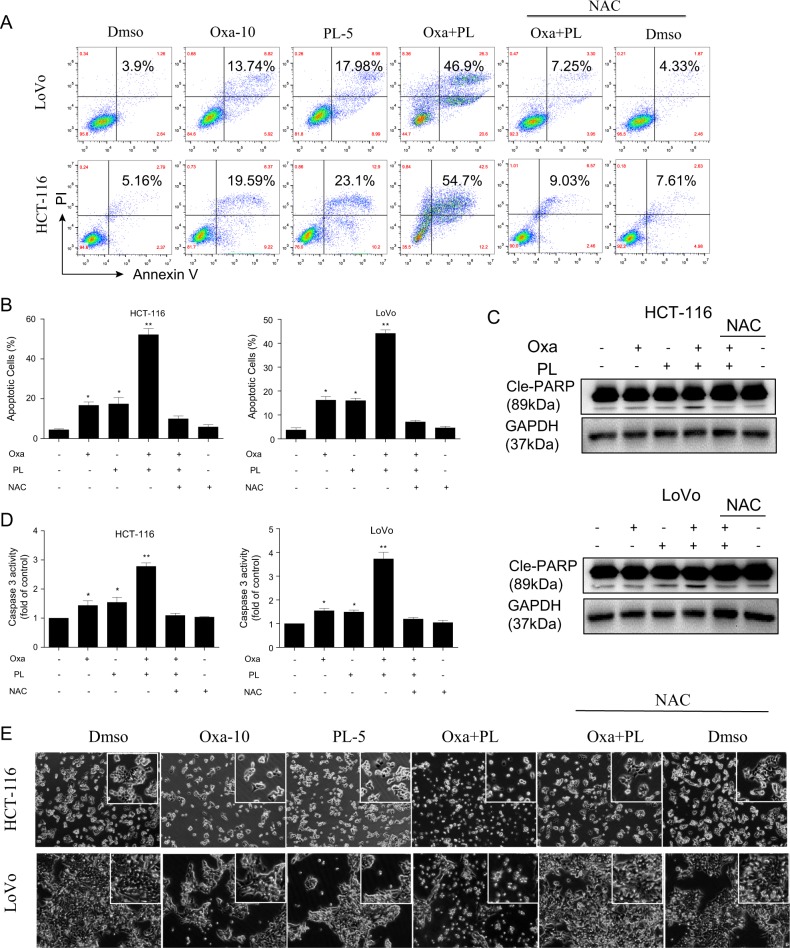
PL increases oxaliplatin-associated cell apoptosis in an ROS-dependent manner. **a** PL enhanced oxaliplatin-associated apoptosis in HCT-116 and LoVo cells (Annexin V/PI staining), and the ROS scavenger NAC prevented colorectal cancer cells from undergoing apoptosis after administration of oxaliplatin/PL combination. **b** Quantitative analysis of **a** [*p < 0.05, **p < 0.01]. **c** Immunoblot assessment of apoptosis-associated proteins after combined administration of PL and oxaliplatin with or without NAC pretreatment. **d** NAC fully reversed caspase-3 activation by combined treatment with PL and oxaliplatin in colorectal cancer cells. The cells underwent preincubation with or without 5 mM NAC for 2 h before oxaliplatin/PL combination administration for 20 h, and caspase-3 activity levels in cell lysates were assessed. **e** Phase contrast micrograph showing that PL significantly enhanced the effects of oxaliplatin on viability and morphology in colorectal cancer cells in an ROS-dependent manner. Sparsely distributed cells with rounded morphology could be seen in HCT-116 and LoVo cells following the combined treatment administered for 24 h, whereas NAC pretreatment fully reversed these changes [scale bar = 20 μm]. Data were from three independent experiments

The association of oxidative stress with apoptosis induced by the combined treatment was next assessed. Thus, the proapoptotic effects of PL/oxaliplatin combination were examined by the Annexin V/PI double staining assay. At the concentrations of oxaliplatin and PL assessed, either agent alone only induced a slight increase in apoptosis. Strikingly, the combination of oxaliplatin and PL at the same concentrations induced dramatic increases in apoptotic cell amounts. Pretreatment with NAC (5 mM) completely blunted apoptosis associated with PL/oxaliplatin combination in both HCT-116 and LoVo cells (Fig. 3a, b). These findings were confirmed by PARP cleavage (Fig. 3c). In addition, the colony formation assay also demonstrated that PL sensitized CRC cells to oxaliplatin, and NAC pretreatment reversed these synergistic antitumor effects (Fig. S3A). Similar findings were obtained for caspase-3 activity (Fig. 3d). Consistent with the above results, HCT-116 and LoVo cells administered oxaliplatin and PL exhibited distinct cell shrinkage and decreased cell density. Meanwhile, NAC pretreatment fully abolished the morphological changes induced by the combined treatment (Fig. 3e). Similar morphological changes, including nuclear condensation and fragmentation, were found in HCT-116 and LoVo cells by Hoechst 33258 staining (Fig. S3B). Collectively, these results indicated PL enhanced oxaliplatin-associated apoptosis via a ROS-mediated signaling pathway.

### PL and oxaliplatin combination activates ER stress and mitochondrial dysfunction

**Fig. 4:**
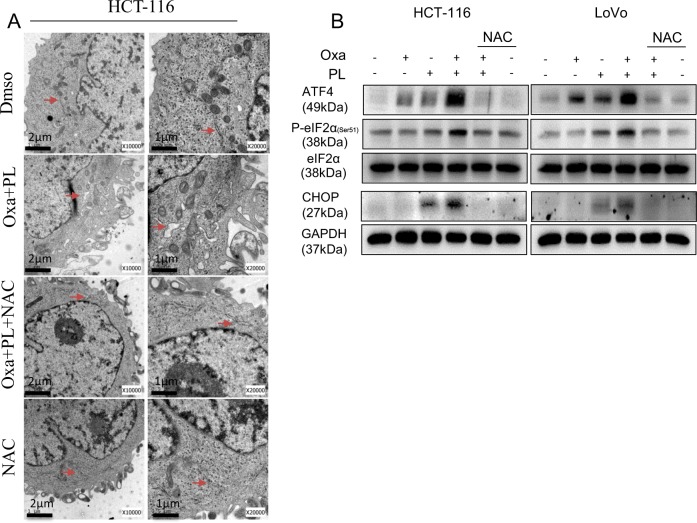
ER-stress response induced by oxaliplatin and PL treatment. **a** The effects of oxaliplatin and PL combination on endoplasmic reticulum (ER) in HCT-116 cells were determined by electron microscopy. HCT-116 were incubated with 10 µM oxaliplatin and 5 µM PL in combination for 6 h with or without preincubation in presence of 5 mM NAC [×10,000 or×20,000]. **b** Immunoblot assessment of ER-stress pathway proteins after combined administration of oxaliplatin and PL for 6 h (p-eIF2α, phosphorylated eukaryotic initiation factor 2α; ATF4, activating transcription factor-4) or 12 h (CHOP, CCAAT/enhancer-binding protein homologous protein). NAC pretreatment for 2 h totally reversed ER-stress induction by oxaliplatin/PL combination. The eIF2α and GAPDH proteins constituted internal controls. Data were from three independent experiments

Raised ROS amounts and unbalanced intracellular redox status increase the amounts of unfolded proteins and promote ER-stress response^16^. Based on the above, we hypothesized that ER-stress exacerbation participates in CRC cell apoptosis induced by oxaliplatin and PL co-administration. Therefore, the levels of the ER-stress-associated proteins eIF2α, ATF4, and CHOP were assessed. As shown in Fig. 4b, p-eIF2α, ATF4, and CHOP amounts were significantly elevated after administration of oxaliplatin/PL combination compared with monotherapy (either oxaliplatin or PL alone). Importantly, pretreatment with NAC markedly blunted the combination’s effects (Fig. 4b). The effect of combined treatment on ER morphology in HCT-116 cells was next assessed by electron microscopy. In comparison with control (DMSO-treated) HCT-116 cells, the ER in HCT-116 cells 6 h upon administration of oxaliplatin and PL showed swelling, indicating that misfolded proteins were accumulated in the ER (arrow, Fig. 4a). Such affect was absent upon NAC pretreatment. These findings suggested the combined treatment activated ROS-dependent ER stress in CRC.

**Fig. 5:**
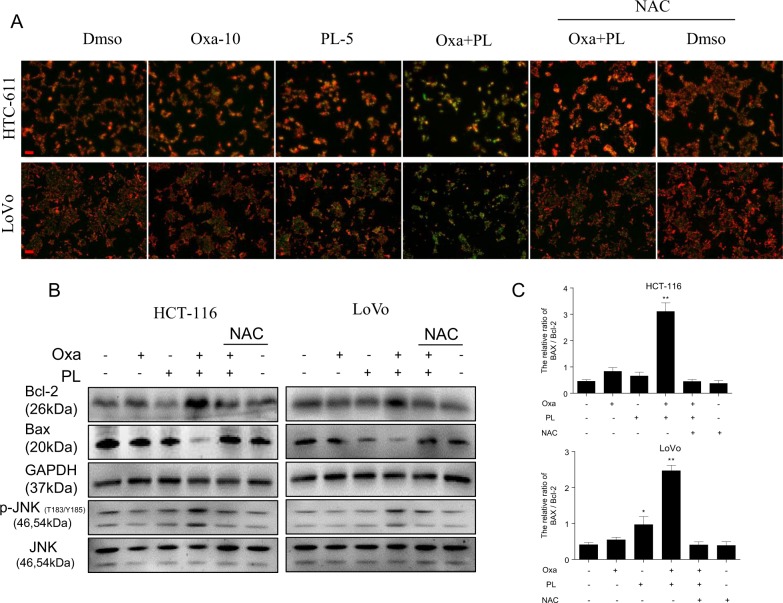
Mitochondrial dysfunction is induced by oxaliplatin and PL treatment. **a** Mitochondrial membrane potential (Δψm) was assessed using the JC-1 dye. Cells were administered 10 µM oxaliplatin and 5 µM PL in combination for 12 h with or without 2 h preincubation with NAC, and submitted to JC-1 staining. Mitochondrial dysfunction was absent in cells administered with DMSO (control), NAC or oxaliplatin, and PL after preincubation with NAC [red, JC-1 accumulation in the mitochondria; green, nonaccumulating JC-1; scale bar = 20 μm]. **b** Treatment of cells with a combination of 10 µM oxaliplatin and 5 µM PL resulted in reduced amounts of the antiapoptotic protein Bcl-2 and elevated levels of the proapoptotic protein Bax, eventually leading to JNK phosphorylation. NAC markedly reversed mitochondrial apoptotic pathway induction by combined administration of oxaliplatin and PL in HCT-116 and LoVo cells. GAPDH and JNK served as internal controls. Data were from three independent experiments

Loss of mitochondrial membrane potential (MMP or △ψm) is a critical index determining cell apoptosis. The synergistic effect of oxaliplatin and PL on MMP was measured with the JC-1 probe, as described above. In this study, treatment with oxaliplatin or PL alone slightly decreased the MMP, whereas oxaliplatin/PL combination significantly decreased MMP in both HCT-116 and LoVo cells (Fig. 5a). Moreover, such mitochondrial depolarization was directly associated with ROS production, as CRC cells pretreated with NAC remained completely unaffected (Fig. 5a). Mitochondria-mediated cell apoptosis involves alterations of Bcl-2 family members by c-Jun N-terminal kinase (JNK)^26,27^. Therefore, Bcl-2 family proteins associated with oxaliplatin/PL combination-induced apoptosis were assessed. Compared with single oxaliplatin or PL treatment, combined treatment dramatically reduced the amounts of the antiapoptotic protein Bcl-2 while increasing those of the proapoptotic protein Bax in both HCT-116 and LoVo cells (Fig. 5b, c). These results indicated that cell apoptosis promoted by oxaliplatin/PL combination was mediated by JNK activation. Indeed, we found that combined treatment with oxaliplatin and PL significantly enhanced JNK phosphorylation compared with oxaliplatin or PL treatment alone (Fig. 5b). In addition, pretreatment with NAC blunted these effects, confirming their associations with oxaliplatin/PL combination-associated oxidative stress (Fig. 5b, c). Taken together, the above findings demonstrated that oxaliplatin/PL combination induced ROS-dependent mitochondrial dysfunction by regulating Bcl-2 family proteins in CRC cells.

### PL amplifies the therapeutic effect of oxaliplatin in vivo

**Fig. 6:**
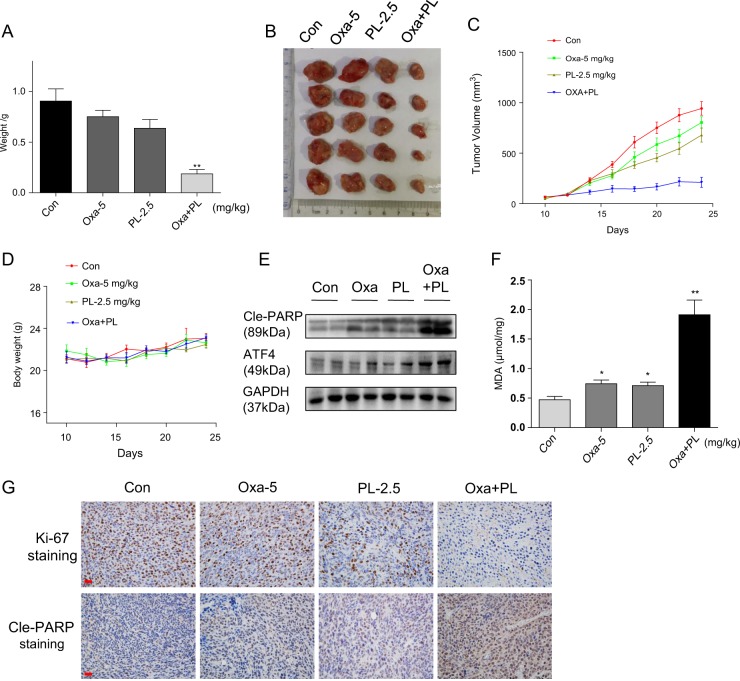
PL enhances oxaliplatin-induced growth inhibition of human colorectal cancer xenografts and induces oxidative injury in vivo. HCT-116 cells were injected into nude mice, which were subsequently administered 5 mg kg^−1^ oxaliplatin and/or 2.5 mg kg^−1^ PL. The combined treatment reduced tumor weight (**a**) and volume (**b** and **c**) [*p < 0.05, **p < 0.01] in nude mice harboring human colorectal cancer xenografts, without affecting animal body weight (**d**). **e** Immunoblot assessment of the expression levels of apoptosis and ER-stress-related proteins (cleaved PARP and ATF4) in tumor specimens. GAPDH served as a reference control. **f** MDA amounts in tumor tissues extracted from xenografts. [*p < 0.05, **p < 0.01]. **g** Immunohistochemical staining of tumor samples for Ki-67 and cleaved PARP, as cell proliferation and apoptosis markers, respectively [scale bar = 50 μm]

Based on the above in vitro findings, the synergistic effects of oxaliplatin and PL on xenograft tumors were examined in vivo. Expectedly, mice injected HCT-116 tumors had reduced xenograft growth after administration of oxaliplatin and/or PL (Fig. 6). Examination of tumors indicated that the monotherapies reduced HCT-116 tumor weights (Fig. 6a), sizes (Fig. 6b and Fig. S5), and volumes (Fig. 6c) to comparable degrees. Meanwhile, the combination therapy had much greater inhibitory effects on tumor growth compared with either monotherapy (Fig. 6a–c). No morphological changes of major organs (e.g., heart, kidney, or liver) were found, and body weights were unaltered, suggesting low or no systemic toxicity of the therapy (Fig. 6d and Fig. S4). To assess whether the mechanisms unveiled in vitro are also involved in vivo, ROS generation and apoptosis were examined in the tumor tissue samples. Immunoblot and immunohistochemistry revealed that oxaliplatin/PL combination significantly elevated ATF4 and cleaved PARP amounts (Fig. 6e, g), indicating that tumor cell apoptosis was associated with ER-stress induction in vivo as well. MDA content represents an adequate index for quantifying cell oxidative stress^28,29^. This study showed that combined treatment with oxaliplatin and PL remarkably enhanced lipid peroxidation (reflected by MDA amounts) in tumor xenografts (Fig. 6f), suggesting elevated ROS biosynthesis and oxidative stress in HCT-116 cell-derived tumors following the combined treatment. Moreover, immunohistochemical staining for Ki-67 in tumor tissue samples revealed Ki-67 positive cells were remarkably reduced by oxaliplatin/PL combination (Fig. 6g). Corroborating in vitro findings, these animal data indicated that PL could sensitize human colon cancer to oxaliplatin in vivo through ROS-mediated cell apoptosis.

## Discussion

Despite advances in the development of diagnostic and treatment tools, CRC remains an important cause of malignancy related death. Oxaliplatin was the first reported platinum containing agent with proven anticancer activity against CRC. It has become the standard treatment for CRC. However, the occurrence of tumor resistance and adverse events still are the major causes of treatment failure^3,30^. Thus, novel therapeutic strategies that would sensitize tumors to oxaliplatin are urgently needed to overcome resistance and toxicity for CRC treatment. The above results corroborated previously published studies. At low treatment dose, colorectal cancer cells show resistance to treatment, while high-dose exert cytotoxic effects on both malignant and normal cells. Thus, combinatorial therapies may be able to offer synergistic anticancer effects with reduced systemic toxicity^31^. Here, the effects of PL, a natural product, in combination with oxaliplatin in human CRC were assessed. We demonstrated that PL sensitized CRC cells to oxaliplatin-associated growth suppression and apoptosis enhancement. In addition, we demonstrated that PL mediated such effects by inducing ROS-dependent ER stress and mitochondrial dysfunction in CRC.

Malignant cells have raised ROS level and antioxidant activities compared with normal cells. Therefore, their higher baseline ROS levels make them more sensitive to products further increasing intracellular ROS^7^. Our previous reports also revealed that synergistic ROS-associated cytotoxicity upon combination therapies are cancer cell-specific^8,15^. In addition, several recent studies have shown antagonism when oxaliplatin is combined with NAC or a superoxide dismutase mimic^12,32^, indicating ROS production plays a crucial role in oxaliplatin cytotoxicity. Our previous work identified PL as a direct TrxR1 inhibitor possessing inhibitory effects on gastric cancer^16^. Therefore, we hypothesized that PL, as a ROS inducer, might constitute a suitable candidate for oxaliplatin-based combinatorial treatment. In the present study, we demonstrated that oxaliplatin dose-dependently induced intracellular ROS production. Furthermore, PL synergistically enhanced oxaliplatin’s anticancer effects on human CRC. To determine the important role of ROS in the effects of oxaliplatin/PL combination, the ROS scavenger NAC was employed. The results showed that NAC fully blunted the synergistic antitumor effects of oxaliplatin and PL on CRC, indicating a ROS-dependent mechanism underlying the combined treatment. Further studies are necessary to reveal the direct redox targets of oxaliplatin as well as the underlying mechanism of ROS generation.

In response to oxidative stress, accumulated unfolded/misfolded proteins trigger ER stress^33^. Normally, ER stress is designed to protect cells as it leads to the production of molecular chaperones and shuts down protein synthesis, whereas increased ER stress causes CHOP-associated cell apoptosis^34,35^. As shown above, oxaliplatin/PL combination increased the amounts of the ER-stress markers ATF4 and p-eIF2α. Moreover, we also confirmed elevated amounts of the ER-stress-specific apoptotic cascade protein CHOP in CRC cells. It should be noted that ROS scavenging by NAC completely eliminated the activation of ER-stress cascade induced by oxaliplatin/PL combination. Collectively, our results suggest the antitumor effects of combined oxaliplatin and PL, at least in part, involve the ROS-associated ER-stress apoptotic cascade.

Raised ROS levels have a significant impact on intracellular processes, including interrupting MMP, cytochrome C release, and mitochondrial function. Activated caspases in turn trigger the proteolytic cleavage of PARP, causing cell apoptosis. The above findings showed combined treatment with oxaliplatin and PL resulted in significantly decreased MMP (Δψm) in CRC cells. Furthermore, ROS scavenging by NAC fully reversed oxaliplatin/PL associated mitochondrial dysfunction, suggesting ROS synthesis might constitute an important upstream regulator of the combined treatment-induced mitochondrial defects. Taken together, the present findings reveal that oxidative stress associated with the combined treatment is directly related to ER stress and mitochondrial dysfunction, amplifying the inhibitory features of combined treatment with oxaliplatin and PL in CRC.

**Fig. 7:**
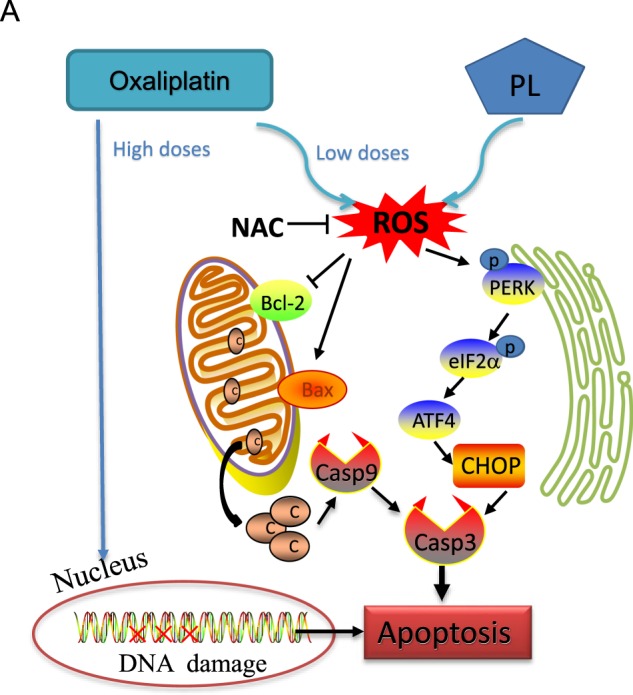
Proposed signaling pathway. Schematic illustration of key findings and the proposed model system. PL enhances oxaliplatin-induced intracellular ROS generation. Increased ROS levels cause ER stress and mitochondria dysfunction, which in turn triggers cell apoptosis

In conclusion, the current study demonstrated PL synergistically increases oxaliplatin’s antitumor activity, revealing a new tool for CRC treatment. Precisely, PL enhanced oxaliplatin’s inhibitory effects on CRC cells mainly through ROS-mediated ER stress and mitochondrial function impairment. Moreover, we confirmed the synergistic anticancer effects of oxaliplatin/PL combination in vivo. Based on these findings, the possible mechanisms behind the synergistic antitumor effects of oxaliplatin and PL on CRC are summarized in Fig. 7. Taken together, we present evidence that oxaliplatin at low concentration combined with PL could represent a potent combinatory regimen for CRC treatment.

## Supplementary information

Supporting information
