# Supplementary material for: The synergistic effects of oxaliplatin and piperlongumine on colorectal cancer are mediated by oxidative stress
Source: Cell Death Dis. 2019 Aug 8;10(8):600. doi: 10.1038/s41419-019-1824-6 (PMC6687721; doi:10.1038/s41419-019-1824-6)
Supplement: Supplementary file 1 — Supporting information [file 41419_2019_1824_MOESM1_ESM.docx]

***Supporting information***

**The synergistic effects of oxaliplatin and piperlongumine on colorectal cancer are mediated by oxidative stress**

WeiQian Chen^1^, WeiShuai Lian^1^, YiFeng Yuan^1^ and MaoQuan Li^1^

^1^Department of Intervention and Vascular Surgery, Shanghai Tenth People’s Hospital, Tongji University School of Medicine, Shanghai, China

**Short title:** Combinatorial oxaliplatin and piperlongumine for colorectal cancer

Corresponding authors:

MaoQuan Li, PhD

Department of Interventional and Vascular Surgery,

Shanghai Tenth People’s Hospital,

Tongji University, Shanghai 200072, China.

E-mail: [lssh2119088@126.com](mailto:lssh2119088@126.com)

**Contents**

Supplemental information includes Methods for supplementary 5 figures.

**Materials for Supplementary data**

**Clonogenic assay**

Cells were seeded at 500 cells/well in 6-well plates and treated with either oxaliplatin, PL, or a combination of the two. Cells were allowed to grow for 7-10 days and stained with crystal violet solution (0.5% in 25% methanol) to count the number of colonies.

**Measurement of reactive oxygen species generation**

Cellular ROS levels were measured by flow cytometry. Briefly, 5×10^5^ cells were plated on 60-mm dishes and allowed to attach overnight. Cells were exposed to either oxaliplatin, PL, or a combination for the indicated time periods. Cells were then stained with 10 μM dichloro dihydrofluorescein diacetate (DCFH-DA; Beyotime Biotech, Nantong, China). DCFH-DA is oxidized to dichloro dihydrofluorescein (DCF) in the presence of ROS. DCF fluorescence was analyzed using FACSCalibur. Meanwhile, the levels of intracellular ROS of cells were also determined by a fluorescence microscope (Nikon, Japan).


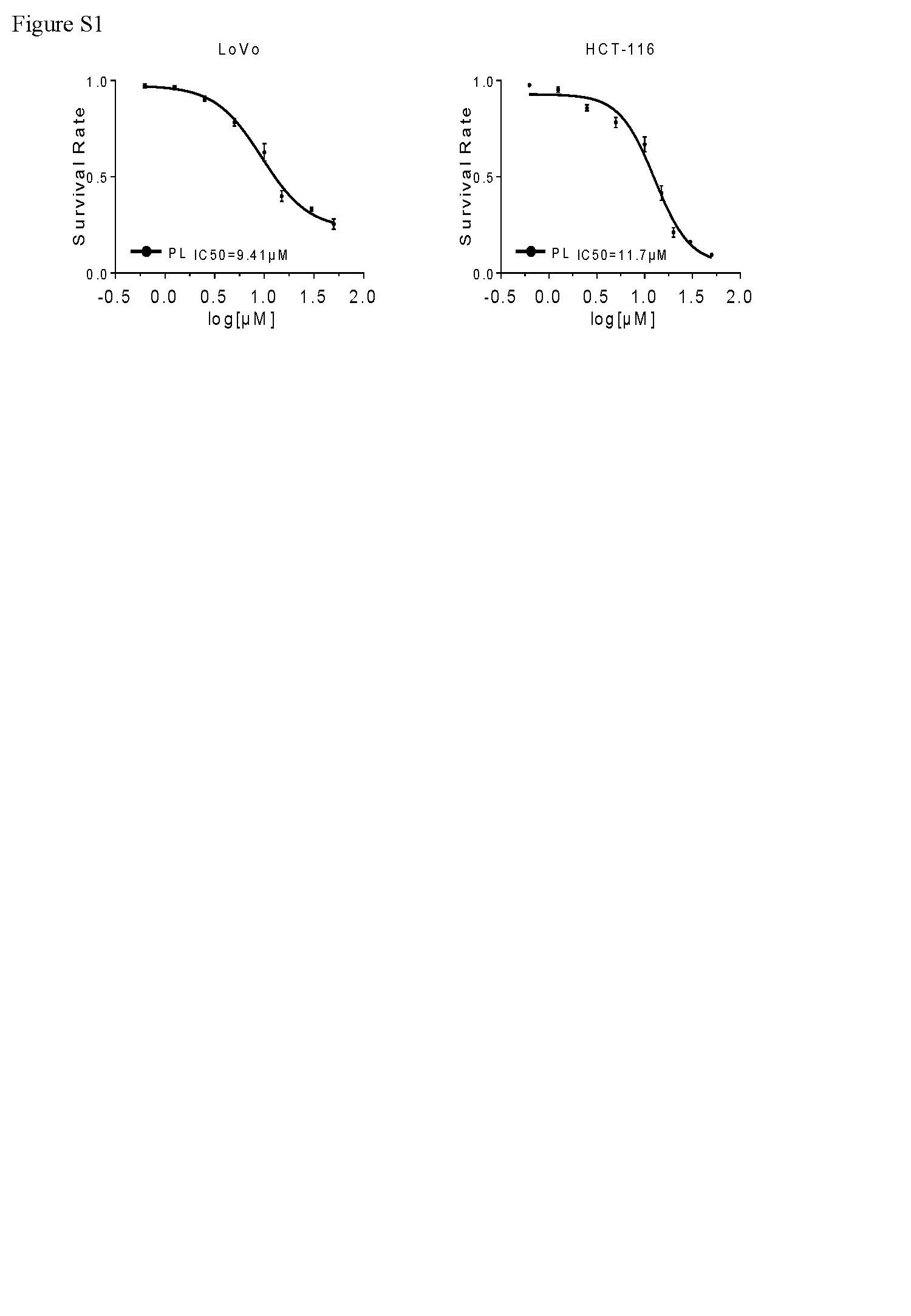


**Figure S1: Effects of PL on viability of human colorectal cancer cells.** The effects of PL on human colorectal cancer cell viability were assessed. The colorectal cancer HCT-116 and LoVo cell lines were administered increasing concentrations of PL (0.625-50μM) for 24 h, and viability was measured by the MTT assay.


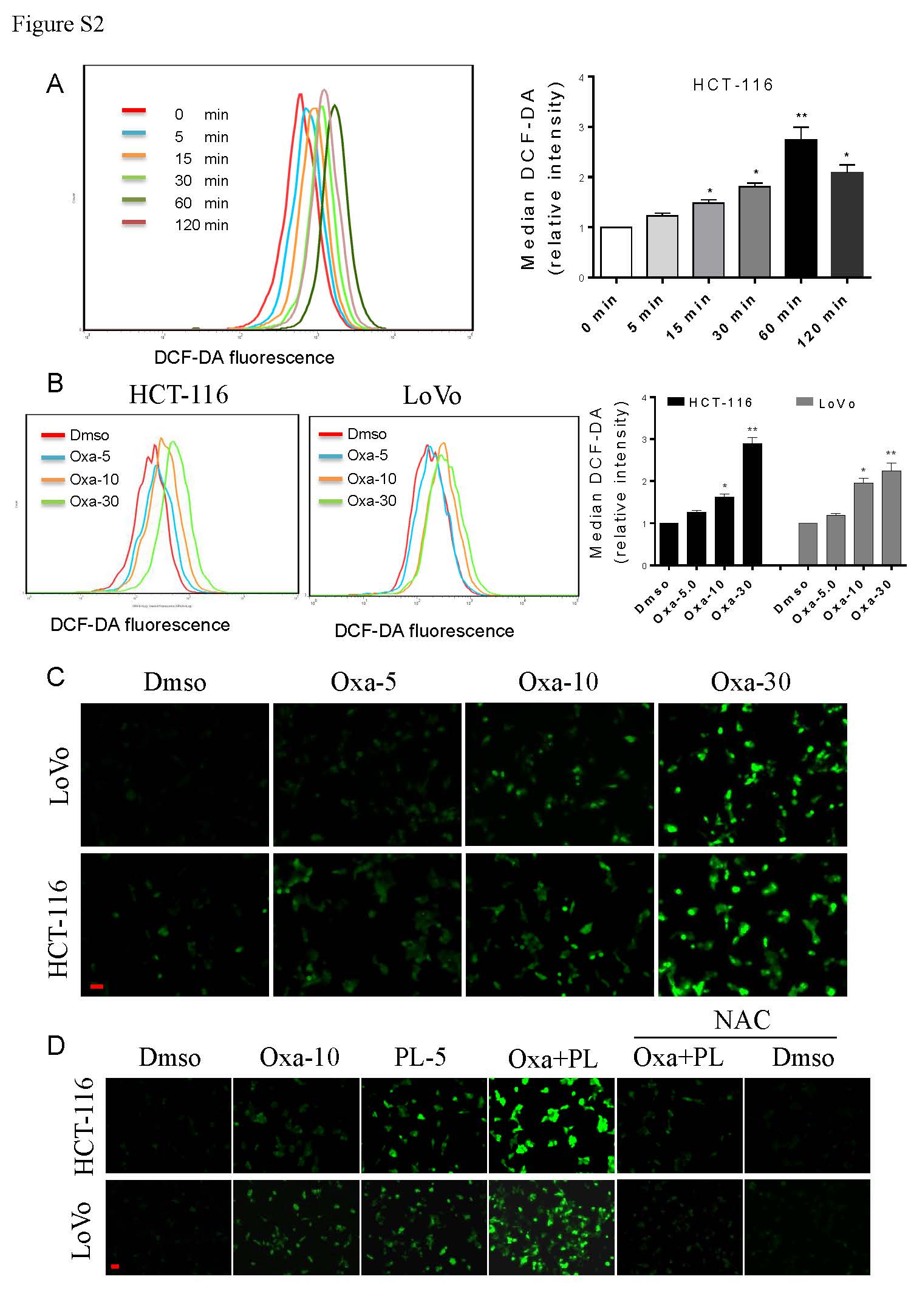


**Figure S2: Oxaliplatin dose-dependently induces ROS generation in human colorectal cancer cells.** (A) Cells were treated with 30μM oxaliplatin alone for the indicated times, and then intracellular ROS amounts were measured using DCFH-DA by flow cytometry. Quantification of DCF flow cytometry data [*p < 0.05, **p < 0.01]. (B and C) Oxaliplatin-induced ROS generation in HCT-116 and LoVo cells. Cells were administered oxaliplatin at the indicated concentrations for 1 h, and then intracellular ROS amounts were assessed using DCFH-DA by flow cytometry and fluorescence microscopy [green = dichlorodihydrofluorescein, DCF; scale bar = 20μm]. (D) Representative DCF fluorescence micrographs of cells administered oxaliplatin and PL with or without NAC pretreatment (green= dichlorodihydrofluorescein, DCF; scale bar = 20μm) from three independent experiments.


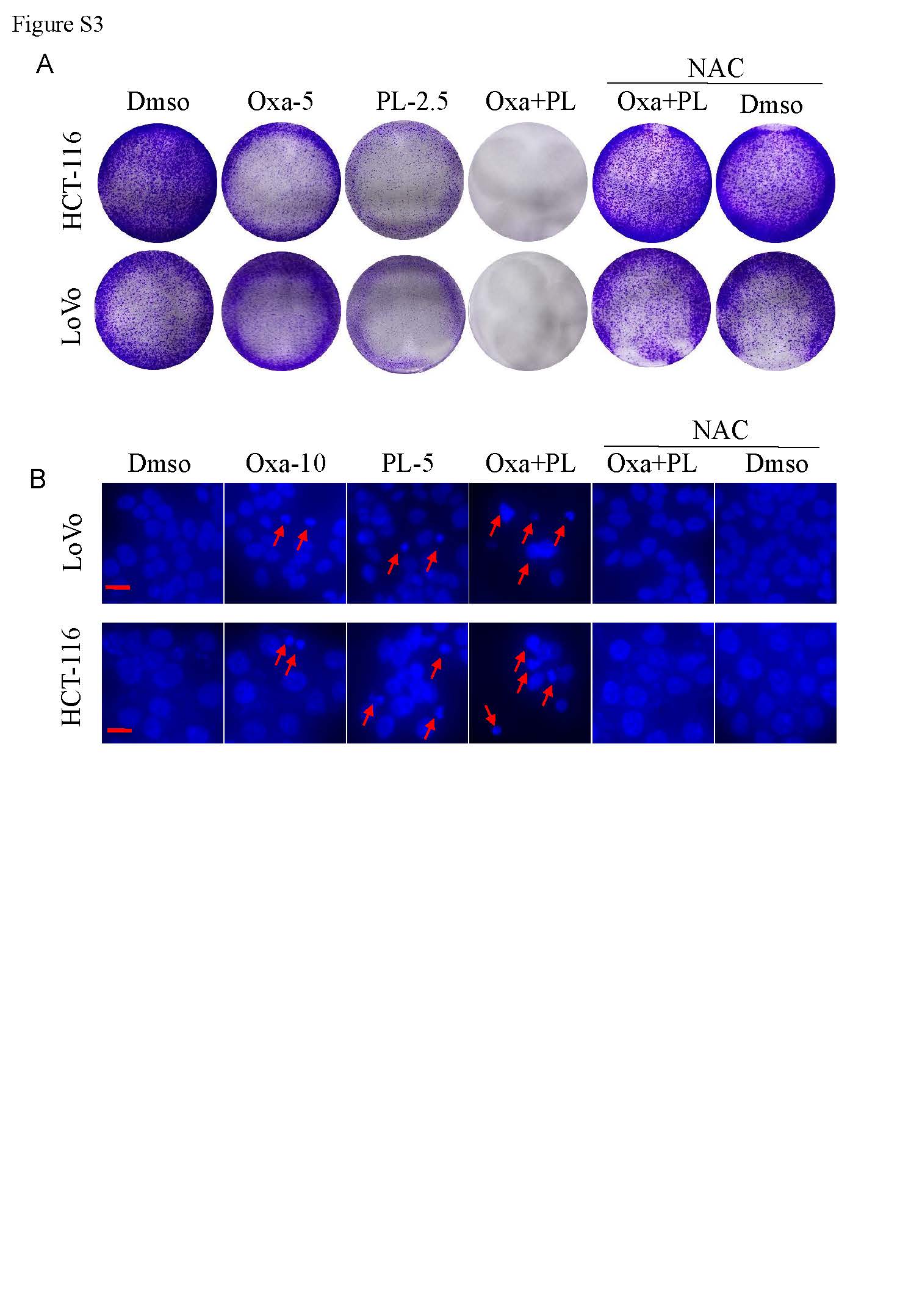


**Figure S3: The synergistic anticancer effects of oxaliplatin and PL involve ROS.** (A) Effects of combined administration of oxaliplatin and PL with or without pre-incubation with NAC on colorectal cancer cell colony formation. Cells were stained with crystal violet after 14 days of treatment. (B) Combined administration of oxaliplatin and PL ROS-dependently induced apoptotic morphology in HCT-116 and LoVo cells. HCT-116 and LoVo cells were administered 10μM oxaliplatin and/or 5μM PL for 12 h with or without NAC pretreatment. Cell morphology was observed under an inverted microscope after Hoechst 33258 staining.


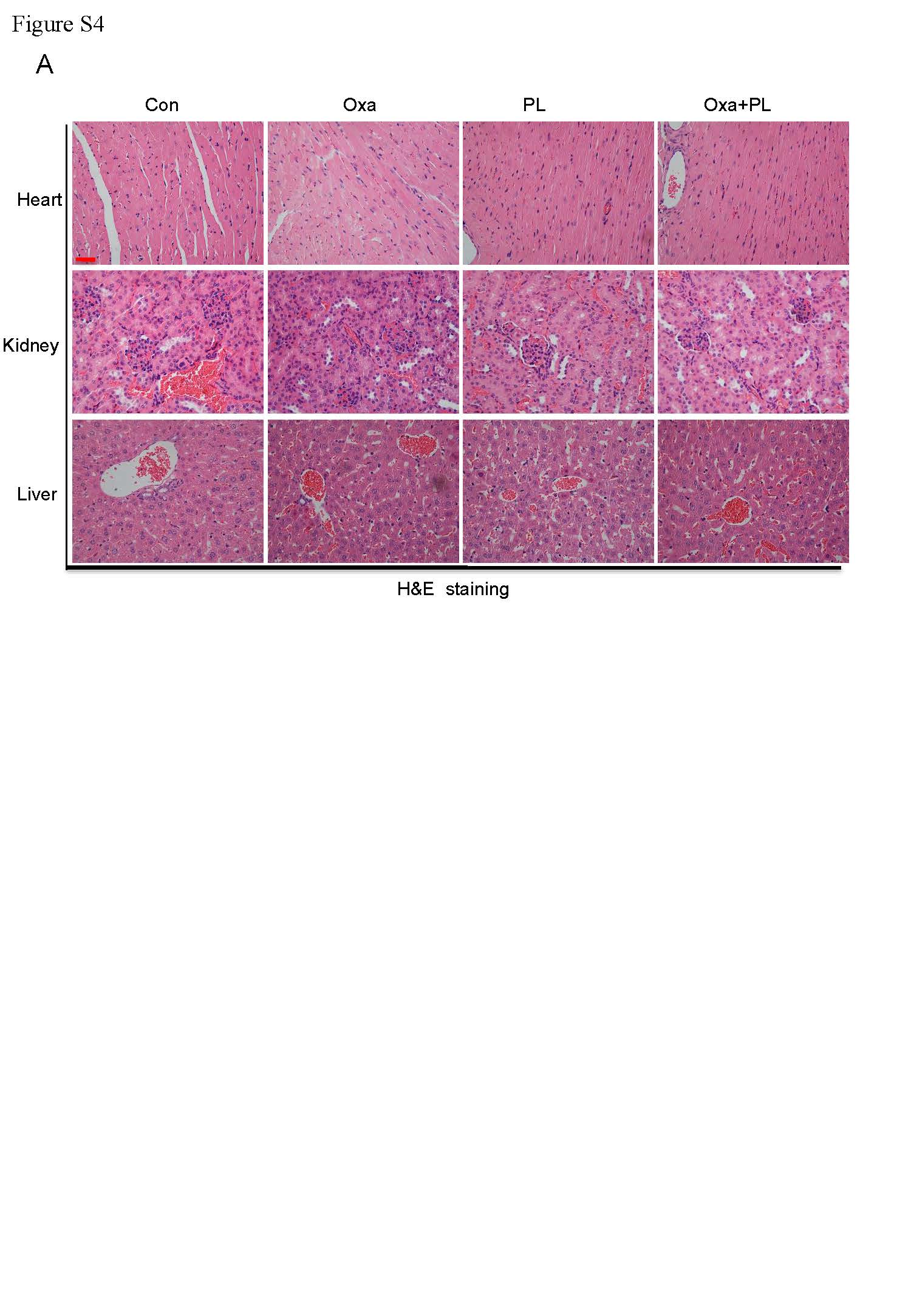


**Figure S4: Oxaliplatin and PL combination causes no overt heart, kidney and liver toxicities in mice.** Hematoxylin and eosin (H&E) staining of kidney, liver, and heart tissue samples from mice treated with oxaliplatin and/or PL [n=5].


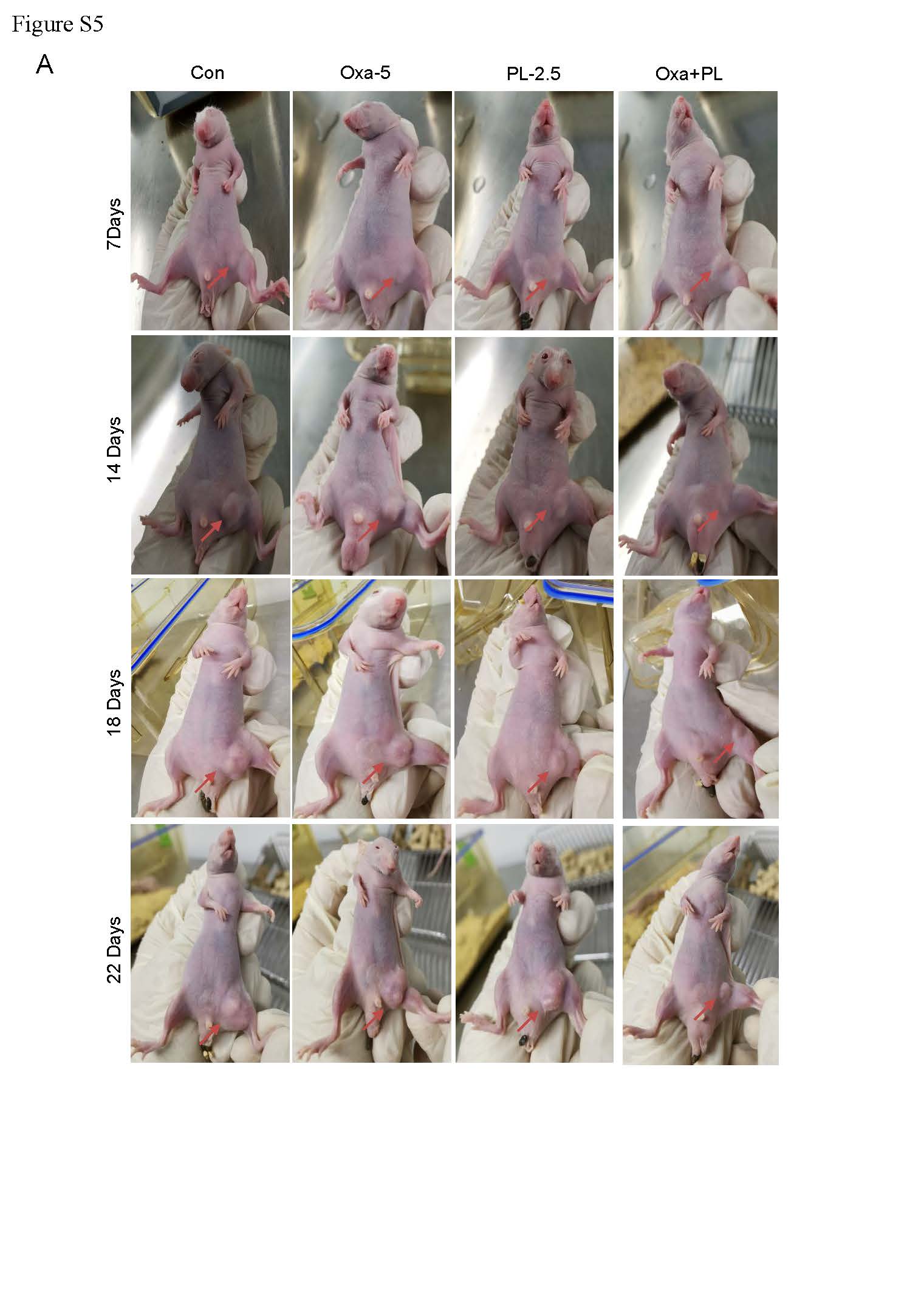


**Figure S5: PL enhances the anti-tumor activity of oxaliplatin in human colorectal cancer xenografts**. Representative images of HCT-116 xenograft tumor from each group dynamically showing tumour size changes at the indicated time [n=5].
